# Complete Genome Sequence of Isolate Bari 1, a Mild Strain of Cauliflower Mosaic Virus

**DOI:** 10.1128/MRA.00534-21

**Published:** 2021-07-08

**Authors:** Kazusato Ohshima, Rikako Ishibashi, Shusuke Kawakubo

**Affiliations:** a Laboratory of Plant Virology, Department of Biological Resource Science, Faculty of Agriculture, Saga University, Saga, Japan; b The United Graduate School of Agricultural Sciences, Kagoshima University, Kagoshima, Japan; DOE Joint Genome Institute

## Abstract

We present here the complete genome sequence of isolate Bari 1, a mild strain of cauliflower mosaic virus (CaMV). The isolate was collected from Diplotaxis tenuifolia (perennial wall-rocket) in Bari, Italy. The genome was 8,020 nucleotides long and shared ≤85.4% nucleotide identity with other CaMV isolates.

## ANNOUNCEMENT

*Cauliflower mosaic virus* (CaMV) is the type member of the genus *Caulimovirus* in the family *Caulimoviridae* (1). A mild strain of CaMV, isolate Bari 1, was collected from Diplotaxis tenuifolia (perennial wall-rocket) in Bari, Italy, before 1977 (2, 3). In this study, we determined the full genome sequence of isolate Bari 1 and compared its properties with the reported CaMV genome sequences.

Bari 1-infected turnip leaves were stored in a deep freezer (Institute of Molecular Cell and Systems Biology, University of Glasgow, UK) until use. Isolate Bari 1 was sap inoculated to Brassica rapa cv. Hakatasuwari plants using 0.01 M potassium phosphate buffer (pH 7.0) and serially cloned through single lesions using chlorotic local lesions that appeared ∼20 days after inoculation. Biologically cloned Bari 1 isolate was propagated in *B. rapa* plants. Viral DNAs were extracted from the infected leaves using the DNeasy plant minikit (Qiagen KK) and amplified using high-fidelity Platinum *Pfx* DNA polymerase (Invitrogen). We carefully sequenced three overlapping fragments of the Bari 1 genome, amplified by PCR using three sets of primer pairs (Table 1). Each PCR product was sequenced by primer walking in both directions using a BigDye Terminator v3.1 Cycle Sequencing Ready Reaction kit (Life Technologies) and an Applied Biosystems 3130 Genetic Analyzer. The sequences were assembled using BioEdit v5.0.9 (4). All tools were run with default parameters unless otherwise specified.

**TABLE 1:** Primers used for amplifying PCR fragments

Primer^*a*^	Type	Sequence (5′–3′)^*b*^	Position (nt)^*c*^	Coding region^*d*^
C10P	Forward	5′-AGTTCCCTCACACCGGTGACC-3′	112–132	ORF VII
C22M	Reverse	5′-TGTCGATTAGGACATTCGTTGG-3′	3503–3524	ORF IV
C17P	Forward	5′-GTAGGAAATGAAGAATTAGGATC-3′	2126–2148	ORF III
C31M	Reverse	5′-GAGCCGTTTGCTCTGGAATAGC-3′	5988–6009	ORF VI
C30P	Forward	5′-AAATCCRAAGATAAGATTCCCA-3′	5693–5714	NCR
C81M	Reverse	5′-CGTCTTCTAGTTCAATTGTAGC-3′	1082–1103	ORF I

a The PCR fragments were amplified using the following three sets of primer pairs: C10P and C22M, C17P and C31M, and C30P and C81M.

b R indicates a mixture of A/G nucleotide degeneracy.

c The nucleotide positions correspond to those of the Xinjiang isolate genome (GenBank accession number AF140604).

d ORF, open reading frame; NCR, noncoding region.

The full genome sequence of isolate Bari 1 was 8,020 nucleotides (nt) long. The lengths of open reading frames (ORFs) I, II, III, IV, V, VI, and VII were 984 nt, 480 nt, 384 nt, 1,476 nt, 2,019 nt, 1,569 nt, and 291 nt, respectively. The start codons in these ORFs were confirmed using the previously reported CaMV genome sequences. The GC content in the Bari 1 genome was 40.1%. The genomic regions of isolate Bari 1 were assessed for nucleotide identity with horseradish latent virus (HRLV) because Bari 1 formed a single lineage distinct from all the CaMV lineages and close to HRLV in the ORF VI phylogenetic tree (5). The reverse transcriptase (RT) and RNase H1 (RH) regions in ORF V of isolate Bari 1 shared 79.1% and 91.1 to 92.7% nucleotide identity, analyzed using EMBOSS Needle (https://www.ebi.ac.uk/Tools/psa/emboss_needle/) (6), with those of HRLV isolate ID1 (GenBank accession number NC_018858) and other CaMV isolates, respectively. The Bari 1 genome shared 67.1% nucleotide identity with the HRLV ID1 genome. Although Bari 1 is biologically distinct from other CaMV isolates, the demarcation criteria for nucleotide identities of species (7) showed that this isolate is a member of CaMV.

**FIG 1:**
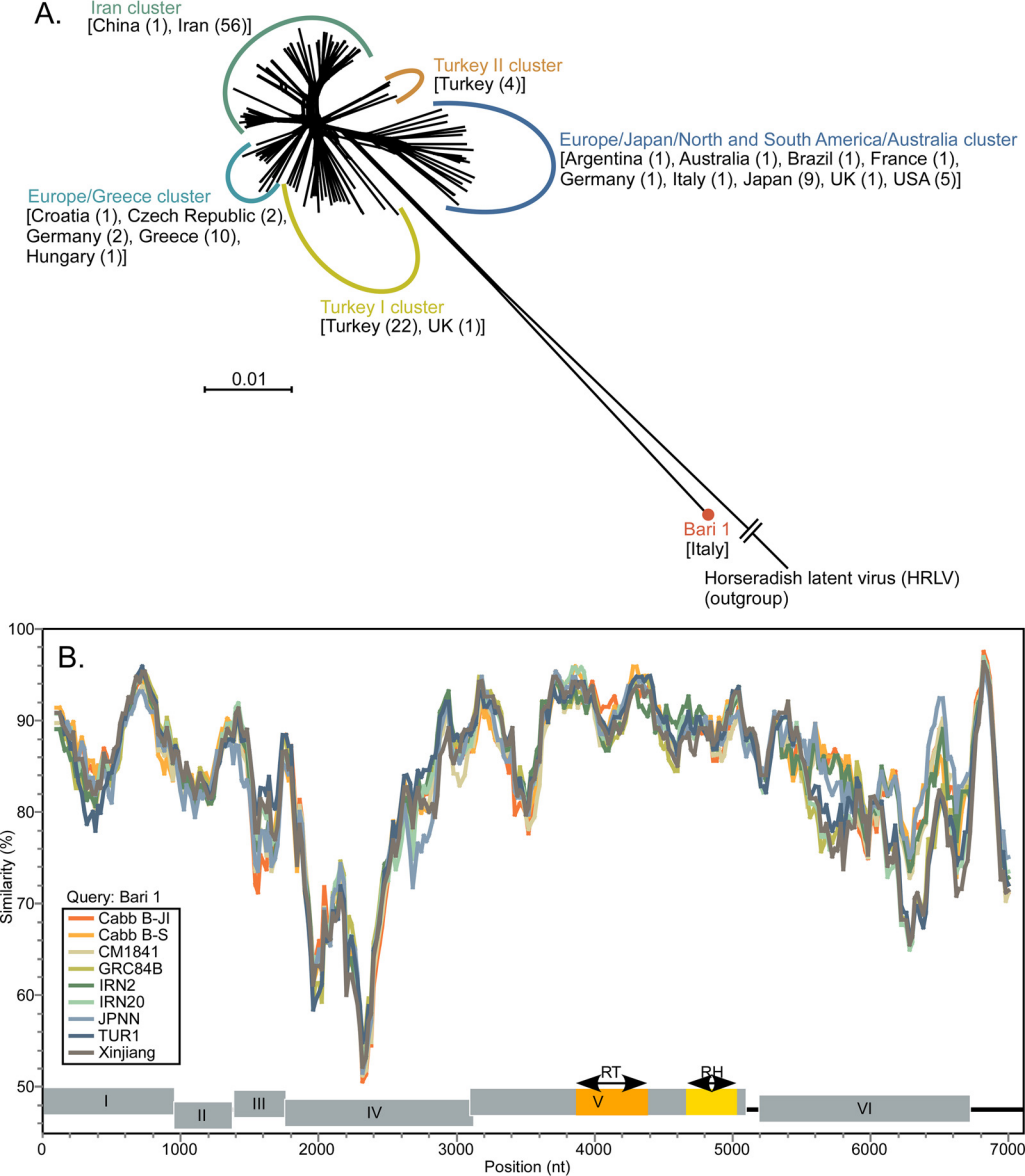
(A) Phylogenetic networks of cauliflower mosaic virus. Neighbor-net analysis inferred from concatenated CaMV ORF regions I to V was performed using SplitsTree v4.15.1 (8). Horseradish latent virus isolate ID1 (GenBank accession number NC_018858) was used as the outgroup taxon. The genomic sequences of 121 CaMV isolates obtained from GenBank (January 2019) and isolate Bari 1 were used. The values given in parentheses are the numbers of isolates from each country. (B) Similarity plot of the nucleotide sequences of CaMV genomes. Isolate Bari 1 (LC632935) was used as the query isolate. The following CaMV isolates were included: Cabb B-JI (KJ716236), Cabb B-S (NC_001497), CM1841 (V00140), GRC84B (AB863194), IRN2 (AB863137), IRN20 (AB863155), JPNN (AB863160), TUR1 (AB863166), and Xinjiang (AF140604). All protein coding regions of CaMV and HRLV were aligned via corresponding amino acid sequences using CLUSTAL X2 (10) with TransAlign (11). After the gaps were removed in each ORF and noncoding region, those sequences were reassembled to form concatenated genome sequences. Overlapping sequences between ORF I and ORF II (3 nt), ORF III and ORF IV (24 nt), and ORF IV and ORF V (36 to 45 nt) were removed. Finally, the concatenated genome sequences of 7,103 nt were used for analysis with SimPlot v3.5.1 (9). Nucleotide similarities were estimated using the Kimura (two-parameter) model, a window size of 200 nt, and a step size of 20 nt.

Network analysis inferred from concatenated ORFs I to V (Fig. 1A) was performed using SplitsTree v4.15.1 (8). The network of CaMV isolates, except Bari 1, had short internal branches. The results of a SimPlot v3.5.1 (9) analysis using the Bari 1 genome sequence as the query isolate are shown in Fig. 1B. The nucleotide similarities between the 5′ half of the ORF IV region of Bari 1 and those of the other CaMV isolates were the lowest. The genome of Bari 1 shared ≤85.4% nucleotide identity with those of the CaMV isolates. This study reports the first complete genome sequence of isolate Bari 1 and confirms that Bari 1 is biologically distinct but a member of CaMV.

### Data availability.

The complete genome sequence of CaMV isolate Bari 1 has been deposited in DDBJ/ENA/GenBank under the accession number LC632935.
